# A New Method of Producing Local Anæsthesia

**Published:** 1873-09

**Authors:** A. Horvath


### SELECTED ARTICLES.

### ARTICLE IV.

### *A New Method of Producing Local Anæsthesia.*

The interest that has been recently manifested in the profession on the subject of anæsthetics, induces us to take an early opportunity of directing our readers to an important paper, by A. Horvath, of Kieff, published in the *Centralblatt fur die Medicinischen Wissenschaften*, proposing a new method of producing local anæsthesia. It is a well-known fact, that if the hand be immersed for a short time in ice-water, an intolerable pain is caused, and the hand has to be withdrawn. In the course of a series of experiments, made in reducing the temperature of frogs by means of cold alcohol, Dr. Horvath observed that no such pain was produced when the hand was immersed in cold alcohol, not even when the temperature was as low as—5° C.

Pursuing the experiment still further, glycerine was found to possess a property similar in this respect to alcohol. Ether, on the other hand, caused pain, the same as ice-water, while the pain produced by cold quicksilver was more acute, causing the speedy withdrawal of the finger when plunged into this liquid at a temperature of -3°. It was next ascertained that, when the finger was held for quite a long time in alcohol having a temperature of -5° C., no pain whatever was experienced, and what was a still more remarkable phenomenon, although the faintest touch was distinctly perceived in this finger, yet no pain whatever was experienced from sharp pricks, which in other fingers were sufficient to cause considerable pain. This experiment seemed to show that the application of cold alcohol has the effect of depriving the part of the special sensibility to pain, without, however, impairing the delicacy of the general tactile sensation, which, as is well known, resides in the superficial integument. This apparent possibility of the artificial separation of these two nervous functions, viz., the tactile sensation, and the sensation of pain, and the temporary suspension of the latter, seemed important in a physiological point of view, and also of no small practical utility in allaying certain forms of local pain, more especially that caused by burns, and surgical operations. With regard to burns, Dr. Horvath soon had an opportunity of testing the value of this application on his own person, as well as upon others, and with the most satisfactory results. Not only was all pain instantly allayed, directly the part was immersed in alcohol, but it was found that the wound very speedily began to assume a more healthy appearance, the surrounding redness rapidly failing. The process of healing seemed to be accelerated. If that theory is a correct one which ascribes the frequent termination of burns to the result of the constitutional shock induced by the severity of the pain, in that case the application of cold alcohol, in that it affords the patient an immediate relief from his sufferings, will prove a powerful agent in such accidents in saving life. In like manner, this same application may be found valuable, it is thought, in cases of traumatic tetanus. The method of producing local anæsthesia by the aid of ice, ether and rhigolene has been perfectly understood for many years. These agents have never been extensively employed, however, inasmuch as it has been found by experience that the process of freezing the part is often productive of quite as serious pain as would have been experienced from the operation without the administration of any anæsthetic. The ether spray is found to be a source of embarrassment to the operator, for, if not carefully directed, it is liable to take effect upon his own fingers, bringing on a sudden numbness, which is more surprising than gratifying. It can, moreover, be applied to only a limited extent of surface at a time.

The extreme simplicity of this new anæsthetic, the ease with which it can be applied to any part of the body where pain is experienced, or when it is desired to make an incision —all these circumstances tend to make it highly probable that its employment will ultimately become general, thereby doing away, in a great measure, with the disagreeable and dangerous effects of ether and chloroform.—*Boston Med. and Surg. Journal.*

